# The effect of the low stromal ratio induced by neoadjuvant chemotherapy on recurrence patterns in borderline resectable pancreatic ductal adenocarcinoma

**DOI:** 10.1007/s10585-021-10142-7

**Published:** 2022-01-09

**Authors:** Kenji Kawahara, Shigetsugu Takano, Katsunori Furukawa, Tsukasa Takayashiki, Satoshi Kuboki, Masayuki Ohtsuka

**Affiliations:** Department of General Surgery, Graduate School of Medicine, Chiba University, 1-8-1, Inohana, Chuo-ku, Chiba City, 260-8677 Japan

**Keywords:** Neoadjuvant chemotherapy, Pancreatic ductal adenocarcinoma, Liver metastasis, Stroma, Gemcitabine plus nab-paclitaxel

## Abstract

The optimal regimens of neoadjuvant chemotherapy (NAC) and its biological and physiological modification of the tumor microenvironment (TME) in patients with borderline resectable pancreatic ductal adenocarcinoma (BR PDAC) remain unknown. A deeper understanding of the complex stromal biology of the TME will identify new avenues to establish treatment strategies for PDAC patients. Herein, we sought to clarify whether stromal remodeling by NAC affects recurrence patterns and prognosis in BR PDAC patients. We retrospectively analyzed data from 104 BR PDAC patients who underwent pancreatectomy with or without NAC (upfront surgery [UpS], n = 44; gemcitabine + nab-paclitaxel [GnP], n = 28; and gemcitabine + S-1 [GS], n = 32) to assess the correlations of treatment with early recurrence, the stromal ratio, and Ki-67 levels. Eighty-six patients experienced recurrence, and those with liver metastasis had significantly shorter recurrence-free survival than those with other recurrence patterns. The frequency of liver metastasis was significantly higher in patients with a low stromal ratio than in those with a high stromal ratio in the NAC group but not in the UpS group. Patients in the GnP group had significantly higher Ki-67 than those in the GS and UpS groups. A low stromal ratio was positively correlated with high Ki-67 in the NAC group but not in the UpS group. The low stromal ratio induced by NAC promoted early liver metastasis in patients with BR PDAC. Our findings provide new insights into the complexity of stromal biology, leading to consideration of the optimal NAC regimen.

**Supplementary Information:**

The online version contains supplementary material available at 10.1007/s10585-021-10142-7.

## Introduction

Pancreatic ductal adenocarcinoma (PDAC) is characterized by high local invasion and hematogenous dissemination and a high frequency of early recurrence, even after curative resection [1]. Recent advancements in multidisciplinary treatment for PDAC have increased the overall 5-year survival rate to 10% [2, 3]. Neoadjuvant chemoradiotherapy has improved clinical outcomes, allowing patients with borderline resectable (BR) PDAC who undergo pancreatectomy to achieve curative resection and prolong their survival [4–6]. However, the optimal regimens of neoadjuvant chemotherapy (NAC) and its biological and physiological modification of the tumor microenvironment (TME) remain unknown.

Gemcitabine plus nab-paclitaxel (GnP) is associated with improved recurrence-free survival (RFS) and overall survival (OS) compared with gemcitabine alone in patients with metastatic PDAC [7]. Dhir et al. reported a median OS of 28.6 months in patients with resectable or BR PDAC undergoing NAC-GnP followed by surgery [8]. NAC with gemcitabine plus S-1 (the prodrug of 5-fluorouracil) (GS) led to a 2-year OS rate of 55.9% in 101 patients with resectable or BR PDAC in a phase II trial [4]. A previous study of NAC-GS showed acceptable feasibility and a high R0 resection rate [9]. Therefore, these two regimens are anticipated to have good chemotherapeutic effects as NAC for BR PDAC.

The desmoplastic stroma characterizing PDAC protects cancer cells against chemotherapies, promoting cancer cell proliferation and migration. However, recent studies have indicated that experimental depletion of the stroma increases PDAC aggressiveness [10–12]. Moreover, recent clinical trials targeting the stroma showed that stromal depletion strategies fail to improve patient survival [13, 14]. A deeper understanding of the complex stromal biology of the TME will identify new avenues to establish treatment strategies for patients with PDAC.

To elucidate the mechanism underlying stromal remodeling of PDACs induced by NAC, we investigated whether different NAC regimens influenced the tumor stroma and were correlated with recurrence patterns. We first analyzed whether NAC affected the ratio of stroma to cancer cells and whether the stromal ratio affected the time to recurrence. Next, we compared molecular differences affected by different NAC regimens. Our findings will provide new insight into whether NAC tips the balance of stroma to cancer cells, resulting in the acceleration of PDAC progression.

## Materials and methods

### Patients and human tissue samples

This study population consisted of 104 patients with BR PDAC who underwent curative pancreatectomy. Patients with preoperative treatments other than NAC-GnP/GS and macroscopic residual tumor (R2) resection were excluded. Tumor and node categories were classified according to the Union for International Cancer Control 8th edition of the American Joint Committee on Cancer classification criteria. The Evans grading system was utilized to assess the histological chemotherapeutic effects. This cohort study was a retrospective and observational study and was approved by the institutional ethics board of Chiba University Graduate School of Medicine (Ethical approval number #3657). Written informed consent was obtained from all patients. This study was performed in accordance with the principles of the Declaration of Helsinki.

### Neoadjuvant chemotherapy (NAC)

The GS group received gemcitabine intravenously at a dose of 1000 mg/m^2^ on days 8 and 15 and S-1 orally twice daily on days 1–14 at a dose according to body surface area (BSA), as follows: BSA < 1.25 m^2^, 40 mg; BSA 1.25–1.5 m^2^, 50 mg; BSA > 1.5 m^2^, 60 mg [15]. The GnP group received intravenous gemcitabine at a dose of 1000 mg/m^2^ and nab-paclitaxel at a dose of 125 mg/m^2^ on days 1 and 8. For both chemotherapies, one cycle was defined as 3 weeks. Patients underwent three cycles of NAC before surgery.

### Masson's trichrome staining and the evaluation of the ratio of the stromal volume to cancer cells

Tumor stromal volume was assessed by Masson's trichrome staining. Each specimen was examined at 40 × magnification and captured at 2448 × 1920 pixels. Digital images were analyzed using Image-Pro 10 software (Media Cybernetics, Silver Spring, MD, USA). Surrounding tissue and acinar cells were excluded. To measure the density and area of collagen, the number of pixels was counted in six color tones of blue-stained collagen, ranging from light blue to dark blue, such that the darkest collagen was counted six times and the lightest collagen was counted only once. The “stromal ratio” was calculated as follows:

Stromal ratio = sum of blue-stained pixels in the six tones/red-stained pixels.

Wherein the red pixels indicated the cytoplasm.

The average stromal ratio calculated from two foci in each specimen was used as the stromal ratio for that sample. The median stromal ratio was 19.2 (interquartile range [IQR]: 12.7–29.8), and the cutoff value for the stromal ratio for predicting prognosis was set at 25.5 according to receiver operating characteristic (ROC) curve analysis.

### Tumor volume assessment

Radiological tumor volume was assessed by multidetector computed tomography (CT). Patients who had > 35% reduction in tumor volume on CT after NAC were classified as responders, and patients who had < 35% reduction were classified as non-responders according to the ROC curve analysis. The threshold of tumor shrinkage for predicting RFS was identified using ROC curve analysis. Tumor volume was calculated using the formula (length × width^2^)/2 [16].

### Immunohistochemistry (IHC)

Formalin-fixed paraffin-embedded tissue samples were cut into 4-μm-thick slices and deparaffinized. Antigens were activated by autoclaving the slides in citric acid buffer (0.01 mol/L, pH 6.0) at 120 °C for 10 min. The slides were blocked with 3% hydrogen peroxide in methanol for 15 min to inactivate endogenous peroxidase. IHC was performed using the hyper-sensitive polymer method (Dako EnVision + kit; Glostrup, Denmark) for tenascin C (TNC), periostin (POSTN), and Ki-67 according to the manufacturer’s protocol. After blocking, the slides were incubated with the following primary antibodies: anti-human TNC (1:1000 dilution; catalog no. sc-20932; Santa Cruz Biotechnology, Inc., CA, USA), anti-human POSTN (1:2000 dilution; catalog no. RD181045050; BioVender, Brno, Czech Republic), and anti-human MIB-1 (1/500 dilution; catalog no. M7240, Dako) overnight at 4 °C. Slides were counterstained with hematoxylin before dehydration, penetration, and mounting. Two 40 × images from each slide were taken by light microscopy, and the staining intensities of TNC and POSTN were evaluated independently by two investigators, including a pathologist who had no knowledge of the patients’ pathological and clinical characteristics. The staining patterns of TNC and POSTN were scored as follows: low expression, staining intensity and area of the stroma around cancer cells less than that of the islet cells (internal positive control); and high expression, staining intensity and area of the stroma around cancer cells greater than or equal to that of the islet cells.

Three different fields representing the areas of highest Ki-67-positive tumor cell density (hot spots) were captured at 200 × magnification. The median number of Ki-67-positive pancreatic cancer cell nuclei was 949 (IQR: 618.3–1254.5), and the median expression level of Ki-67 was 35.5% (IQR: 18.9%–57.0%). Expression in ≥ 35.5% of tumor cells was regarded as high Ki-67, and expression in < 35.5% of tumor cells was regarded as low Ki-67.

### Statistical analysis

The chi-square test or Fisher’s exact test was performed as appropriate for categorical variables, and the Wilcoxon rank sum test, Wilcoxon signed rank test, or Kruskal–Wallis test was applied for continuous variables. OS and RFS were measured from the date of the first treatment until the date of relapse and death from any cause, respectively. Survival curves were generated using the Kaplan–Meier method, and differences between survival curves were compared using the Wilcoxon test. Hazard ratios and 95% confidence intervals were calculated using Cox proportional hazards regression models. In univariate and multivariate analyses of RFS, all cases were censored at 24 months. Variables with *p* < 0.1 in the univariate analysis were further examined by multivariate analysis. Differences with *p* < 0.05 were considered statistically significant. Analyses were performed using R version 4.0.3 (The R Foundation, Vienna, Austria; http://www.R-project.org).

## Results

### Liver metastasis is the earliest recurrence pattern in patients with BR PDAC treated with NAC and surgery

**Fig. 1:**
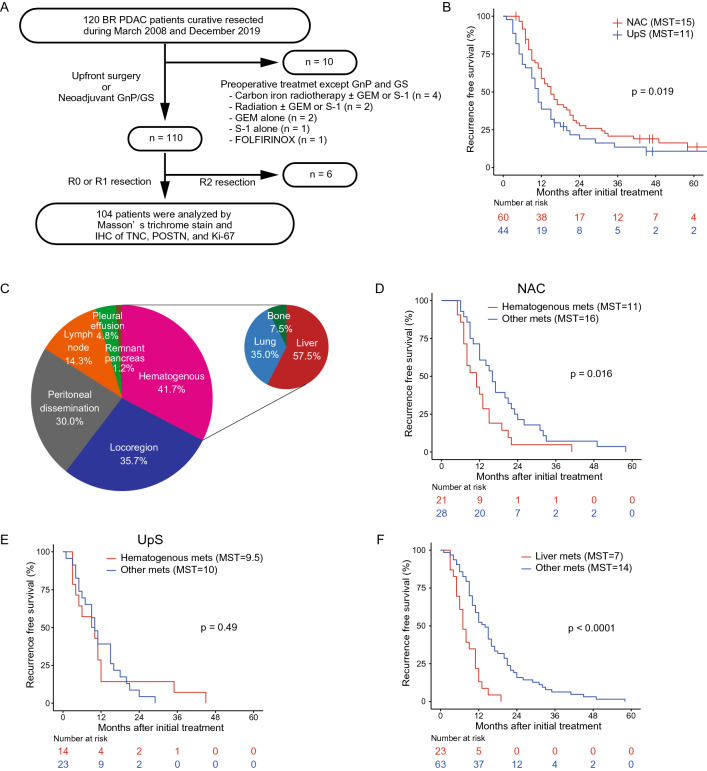
Study profile and effect of NAC on recurrence. **A** Flow chart of patient selection. **B** RFS in the NAC and UpS groups. **C** Initial recurrence patterns (some patients had one initial recurrence at more than one site). **D**, **E** RFS in patients with and without hematogenous dissemination in (**D**) the NAC group and (**E**) the UpS group. **F** RFS in patients with and without liver metastasis. *NAC* neoadjuvant chemotherapy, *RFS* recurrence-free survival, *UpS* upfront surgery

Between March 2008 and December 2019, 120 patients with BR PDAC underwent radical pancreatectomy with or without neoadjuvant treatment in the Department of General Surgery, Chiba University Hospital, Japan. Ten (8.3%) patients who received other neoadjuvant treatments, including carbon ion radiotherapy ± Gem or S-1 (n = 4), radiation ± Gem or S-1 (n = 2), Gem alone (n = 2), S-1 alone (n = 1), and FOLFILINOX (n = 1), and 6 (5%) patients who underwent R2 resection were excluded from the analysis (Fig. 1A). NAC before surgery was administered in 60 (57.7%) patients (NAC group), and 44 (42.3%) patients underwent surgery without NAC (upfront surgery [UpS] group). Patient characteristics in each group are summarized in Table 1. There were some differences in the treatment strategy during the period of this cohort. The initial treatment strategy was UpS for BR PDAC, especially for BR-PV PDAC. NAC-GS was introduced in 2011 based on the preliminary results of clinical trial [9]. NAC-GnP was conducted from 2015 [7]. However, even after 2011, BR PDAC patients with certain complications such as severe renal disfunction, did not receive NAC. The NAC group had a tendency toward a favorable OS (28 months) compared to that in the UpS group (22 months) (*p* = 0.16; Supplementary Fig. S1A), and the median RFS in the NAC group (15 months) was significantly longer than that in the UpS group (11 months) (*p* = 0.019; Fig. 1B). Eighty-six (82.7%) patients experienced disease recurrence after surgery; the initial recurrence patterns varied (Fig. 1C). The administration of NAC is anticipated to control systemic dissemination of PDAC cells; nevertheless patients with hematogenous recurrence developed recurrence earlier than those with other recurrence patterns in the NAC group (*p* = 0.016; Fig. 1D) but not in the UpS group (*p* = 0.49; Fig. 1E). Patients in the NAC group who developed liver metastasis had a significantly shorter RFS (7 months) than those with other recurrence patterns (14 months) (*p* < 0.0001; Fig. 1F), leading to poorer OS (19 months) (*p* = 0.0081; Supplementary Fig. S1B).

**Table 1:** Comparisons of clinicopathological parameters among the UpS, GnP, and GS groups

Clinicopathological parameters	UpS (n = 44)	GnP (n = 28)	GS (n = 32)	*p* value*
Age [IQR]	67.5 [62, 75]	69 [64, 75]	65 [60, 71]	0.45‡
Sex (M/F)	29/15	13 /15	24/8	0.065
Operation (PD/DP/TP)	39/4/1	20/8/0	20/9/3	0.024†
BR-PV/BR-A	25/19	6/22	9/23	0.004
PVR (+/−)	35/9	19/9	18/14	0.093
Arterial resection (+/−)	4/40	3/25	10/22	0.031†
Histological grade (1,2/3,4)	37/7	24/4	29/3	0.76†
pT (1,2/3,4)	31/13	22/6	20/12	0.4
pN (0/1,2,3)	8/36	7/21	6/26	0.76
UICC stage-8th (≤ IIA/ ≥ IIB)	14/30	8/20	7/25	0.63
Evans grade (I/IIa/IIb/III)	NA	8 /1/5/0	15/14/2/1	0.22†
RDI of NAC (mean: %)	NA	97.1	87	0.006§
Adjuvant chemotherapy	5/15/17/7/0	2/25/1/0/0	4/7/2/18/1	< 0.001
(None/S-1/Gem/GS/SC)				

*n* indicates the number of participants, *UpS* upfront surgery, *GnP* gemcitabine plus nab-paclitaxel, *GS* gemcitabine plus S-1, *IQR* interquartile range, *PD* pancreaticoduodenectomy, *DP* distal pancreatectomy, *TP* total pancreatectomy, *PVR* portal vein resection, *NA* not applicable, *RDI* relative dose intensity, *Gem* gemcitabine, *SC* S-1 plus cisplatin

^*^, chi-square test unless otherwise noted; †, Fisher exact test, ‡, Kruskal–Wallis test; §, Wilcoxon rank sum test

### A low stromal ratio is associated with liver metastasis and poor prognosis

**Fig. 2:**
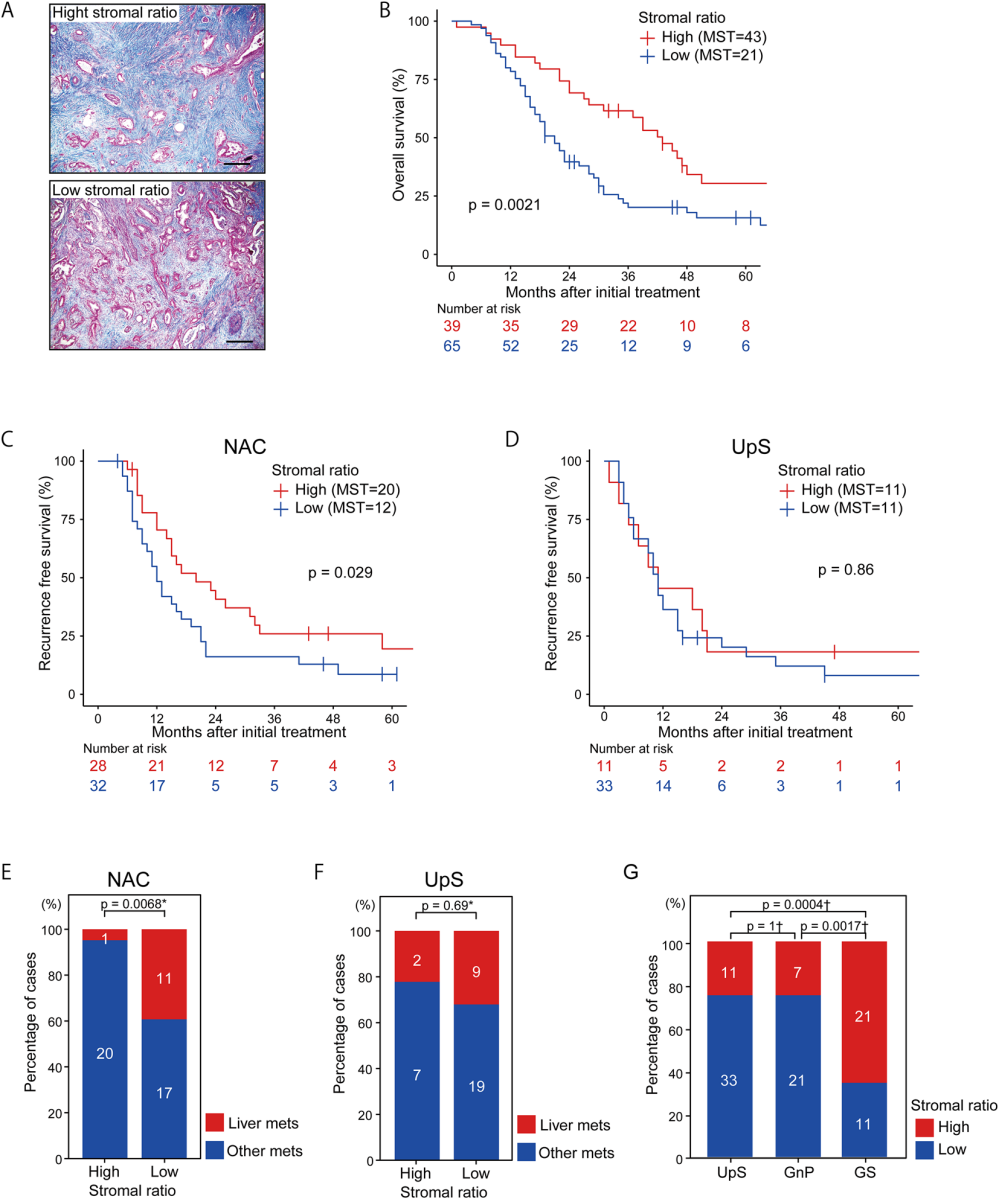
Analysis of stromal ratio. **A** Representative specimens of a (upper panel) high and (lower panel) low stromal ratio. Scale bar: 400 μm. **B** OS according to stromal ratio. **C**, **D** RFS according to stromal ratio (**C**) in the NAC and (**D**) UpS groups. **E**, **F** Liver metastases and other metastases based on the stromal ratio in (**E**) the NAC and (**F**) UpS groups. **G** Comparison of stromal ratio by treatment method. *Fisher exact test, †chi-square test. *OS* overall survival; *RFS* recurrence-free survival, *NAC* neoadjuvant chemotherapy, *UpS* upfront surgery

Experimental studies have demonstrated that the stroma, which is composed of a variety of extracellular matrix (ECM) factors, is a key factor in PDAC progression [17]. Collagens are the most abundant and well-characterized ECM component. To investigate whether the stromal ratio affects recurrence patterns in patients treated with NAC followed by surgery, collagen was quantitatively estimated using Masson's trichrome staining to distinguish tumor cells from surrounding collagenous connective tissue (Fig. 2A, Supplementary Fig. S2A-H). Patients were divided into high and low stromal groups based on their stromal ratio by the ROC curve analysis (Supplementary Fig. S2I). Patients with a low stromal ratio had a significantly shorter OS than those with a high stromal ratio (*p* = 0.0021; Fig. 2B). Interestingly, patients in the NAC group with a low stromal ratio had a significantly shorter RFS than those with a high stromal ratio (*p* = 0.0029; Fig. 2C), whereas no difference in RFS was observed in the UpS group (*p* = 0.86; Fig. 2D). Notably, the frequency of liver metastasis after surgery was significantly higher in patients with a low stromal ratio than in those with a high stromal ratio in the NAC group (*p* = 0.0068; Fig. 2E) but not in the UpS group (*p* = 0.69; Fig. 2F). Furthermore, the GS group had a significantly higher proportion of patients with a high stromal ratio than both the GnP group (*p* = 0.0017) and the UpS group (*p* = 0.0004; Fig. 2G). These data suggest that a low stromal ratio may facilitate liver metastasis, and NAC with S-1 may block stromal depletion during tumor shrinkage.

### Radiological tumor volume does not correlate with the stromal ratio

**Fig. 3:**
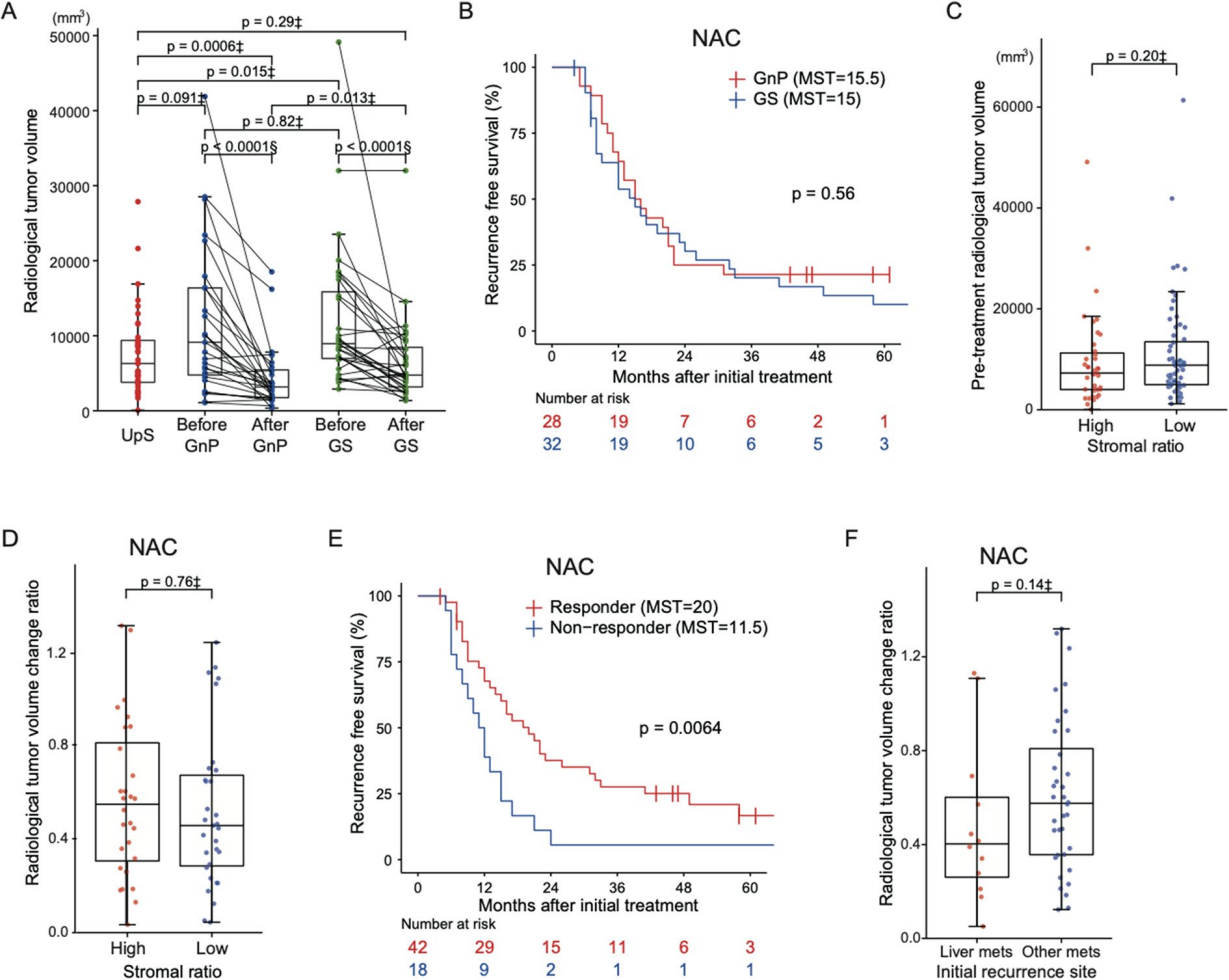
Assessment of radiological tumor volume. **A** Comparison of radiological tumor volume based on the treatment method and changes in the radiological tumor volume before and after treatment with GnP and GS. **B** RFS according to NAC regimen. **C** Correlation between radiological tumor volume and stromal ratio. **D** Correlation between stromal ratio and radiological tumor volume change (tumor volume calculated by CT after NAC divided by that before NAC.) **E** RFS according to tumor shrinkage (patients whose radiological tumor volume shrunk more than 35% after NAC were defined as “responders;” all others were defined as “non-responders”). **F** Correlation between liver metastasis and radiological tumor volume change. ‡Wilcoxon rank sum test, §Wilcoxon signed rank test. *GnP* gemcitabine and nab-paclitaxel, *GS* gemcitabine and S-1, *RFS* recurrence-free survival, *NAC* neoadjuvant chemotherapy, *CT* computed tomography

Because there was a significant association between a low stromal ratio and liver metastasis, we wanted to identify preoperative parameters that could predict the stromal ratio. Therefore, we assessed whether there was a correlation between the stromal ratio and radiological tumor volume. The radiological tumor volume just before surgery was significantly smaller in the GnP group than in the UpS group (*p* = 0.0006) and the GS group (*p* = 0.013) (Fig. 3A). Although the rate of tumor shrinkage in the GnP group was significantly higher than that in the GS group, Kaplan–Meier analysis indicated no difference in the RFS between the GnP and GS groups (*p* = 0.56; Fig. 3B). No positive correlation was observed between the radiological tumor volume and stromal ratio (*p* = 0.20; Fig. 3C), and the rate of tumor shrinkage by NAC was not correlated with the stromal ratio (*p* = 0.76; Fig. 3D). Divided into responder and non-responder groups based on the ROC curve analysis (Supplementary Fig. S3A), 42 (70%) responders had significantly longer RFS than 18 (30%) non-responders (*p* = 0.0064; Fig. 3E). The rate of tumor shrinkage tended to be higher in patients with liver metastasis than in those with other recurrence patterns, but only in the NAC group (*p* = 0.14; Fig. 3F). These findings indicate that radiological “tumor shrinkage” does not represent the actual reduction of cancer cells or the ratio of stroma to cancer cells induced by NAC. Further, the patients in the NAC group were divided into two groups; grade I as low responders and grade IIa or higher as high responders according to the Evans grading system which assesses the pathological volume of residual tumor cells. There was no correlation between the Evans grade and the stromal ratio in this cohort (*p* = 0.15; Supplementary Fig. S3B). We also observed no significant difference of the Evans grade between the GnP and GS groups (*p* = 0.15; Supplementary Fig. S3C).

### A low stromal ratio has a positive correlation with high Ki-67 in patients receiving NAC

**Fig. 4:**
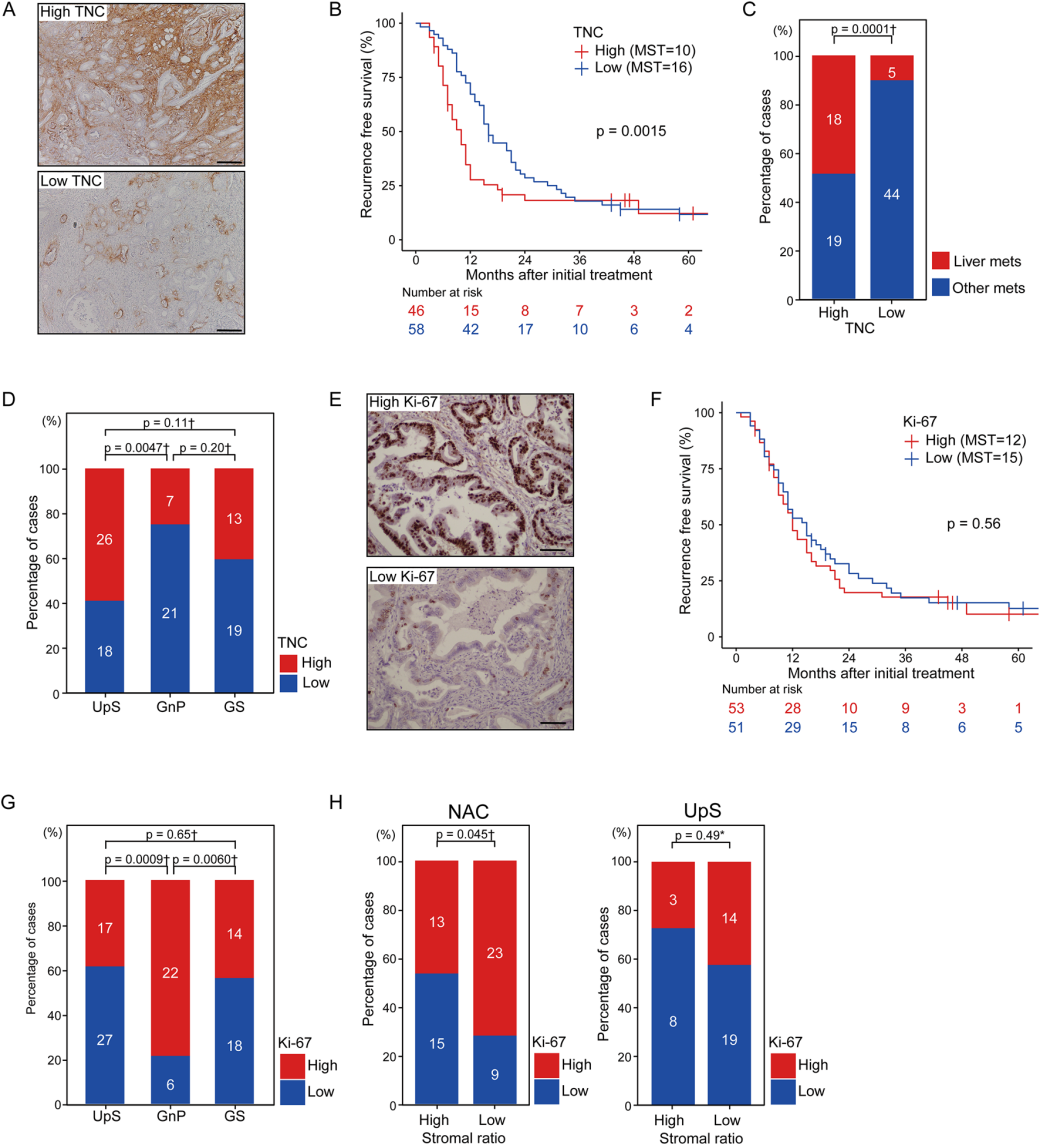
Evaluation of TNC and Ki-67 expression on RFS. **A** IHC analysis was performed using an anti-TNC antibody, and patients were classified into two groups according to TNC expression. Representative examples of high TNC expression (upper panel) and low TNC expression (lower panel). Scale bar: 200 μm. **B** RFS according to TNC expression. **C** Correlation between TNC expression and liver metastasis. **D** Correlation between treatment method and TNC expression. **E** IHC of Ki-67. Representative examples of high Ki-67 expression (upper panel; 547 of the 596 nuclei [91.8%] were positive) and low Ki-67 expression (lower panel: 76 of the 494 nuclei [15.4%] were positive). Scale bar: 50 μm. **F** RFS according to Ki-67 expression. **G** Correlation of treatment method and Ki-67 expression. **H** Correlation of stromal ratio and Ki-67 in the NAC group (left) and the UpS group (right). *Fisher exact test, †chi-square test. *IHC* immunohistochemistry, *TNC* tenascin C, *RFS* recurrence-free survival, *NAC* neoadjuvant chemotherapy, *UpS* upfront surgery

The ECM components of PDAC influence cancer cell proliferation, invasion, and metastasis. To investigate the clinical significance of other factors, two other major components of the stroma that are highly expressed in PDAC, TNC and POSTN, were assessed by immunostaining, and patients were divided into high and low expression groups. Forty-six patients (44.2%) had high TNC expression and 58 (55.8%) had low TNC expression (Fig. 4A). Thirty-six patients (34.6%) had high POSTN expression, and 68 patients (65.4%) had low POSTN expression (Supplementary Fig. S4A). Patients with high TNC expression had a significantly shorter RFS (*p* = 0.0015; Fig. 3B) and a higher frequency of liver metastasis (*p* = 0.0001; Fig. 4C) than those with low TNC expression, whereas POSTN expression was not associated with early recurrence (Supplementary Fig. 4B, C). The expressions of TNC and POSTN were significantly decreased in the GnP group compared to those in the UpS group (Fig. 4D, Supplementary Fig. S4D).

The interaction of stroma and cancer cells fosters cancer progression in PDAC. To better understand the paradoxical finding wherein a greater reduction in radiological tumor volume by NAC increased the tendency of metastasis to the liver, the proliferative status of PDAC cells was assessed (Fig. 4E). Kaplan–Meier analysis did not reveal a significant difference in the RFS based on Ki-67 staining (*p* = 0.57; Fig. 4F), which is consistent with the findings of previous reports [18–20]. Surprisingly, the GnP group had significantly higher Ki-67 than the GS group (*p* = 0.0060) or the UpS group (*p* = 0.0009) (Fig. 4G). Furthermore, a low stromal ratio was positively correlated with high Ki-67 in the NAC group (*p* = 0.045) but not in the UpS group (*p* = 0.36) (Fig. 4H). Finally, we evaluated the clinical parameters that correlated with early recurrence in patients with BR PDAC. LN positivity, portal vein invasion, and a low stromal ratio were identified as independent significant parameters of early recurrence in the multivariate analysis (Table 2). Notably, a low stromal ratio was identified as an independent predictive parameter of early recurrence in the multivariate analysis in the NAC group (*p* = 0.0061; Table 3) but not in the UpS group (Supplementary Table S1). Taken together, these results demonstrate that stromal remodeling by NAC affects stromal depletion, upsets the balance of stroma to cancer cells, and facilitates cancer cell proliferation, leading to early liver metastasis after surgery in patients with BR PDAC.

**Table 2:** Univariate and multivariate analysis of predictors of recurrence-free survival

Clinico-pathological parameters	n	Univariate analysis		Multivariate analysis	
		HR (95% CI)	*p* value	HR (95% CI)	*p* value
Age (< 70/≥ 70) (years)	60/44	1.21 (0.79–1.85)	0.38		
Sex (M/F)	66/38	1.10 (0.71–1.70)	0.67		
Neoadjuvant chemotherapy (− /+)	44/60	1.44 (0.94–2.20)	0.092	1.09 (0.70–1.69)	0.71
PVR (−/+)	37/67	1.90 (1.17–3.08)	0.0099	1.67 (1.01–2.71)	0.044
Arterial resection (− /+)	87/17	0.61 (0.33–1.13)	0.12		
Preoperative CA19-9 (< 70/≥ 70) (U/mL)	33/71	1.37 (0.87–2.16)	0.18		
Lymph node metastasis (−/+)	21/83	2.18 (1.24–3.82)	0.0065	2.25 (1.28–4.01)	0.0074
Histological grade (0,1/2,3)	90/14	0.74 (0.37–1.48)	0.4		
Pre-treatment tumor volume (< 7000/≥ 7000) (mm^3^)	44/60	0.91 (0.60–1.39)	0.66		
Stromal ratio (High/Low)	65/39	1.65 (1.06–2.59)	0.027	1.69 (1.07–2.67)	0.04
TNC (Low/High)	58/46	1.51 (0.98–2.32)	0.06	1.12 (0.76–1.83)	0.36
POSTN (Low/High)	68/36	0.87 (0.56–1.36)	0.55		
Ki-67 (Low/High)	51/53	1.12 (0.73–1.70)	0.61		

The left category is the reference for the hazard ratio

*n* indicates the number of participants, *HR* hazard ratio, *CI* confidence interval, *PVR* portal vein resection

**Table 3:** Univariate and multivariate analysis of predictors of recurrence-free survival in the neoadjuvant chemotherapy group

Clinico-pathological parameters	n	Univariate analysis		Multivariate analysis	
		HR (95% CI)	*p* value	HR (95% CI)	*p* value
Age (< 70/≥ 70) (years)	34/26	1.41 (0.80–2.49)	0.23		
Sex (M/F)	37/23	1.04 (0.59–1.86)	0.88		
Neoadjuvant chemotherapy (GnP/GS)	28/32	1.23 (0.70–2.16)	0.47		
PVR (− /+)	23/37	1.57 (0.87–2.83)	0.14		
Arterial resection (−/+)	47/13	0.80 (0.40–1.60)	0.53		
Preoperative CA19-9 (< 70/≥ 70) (U/mL)	17/43	1.98 (1.04–3.76)	0.037	1.62 (1.02–2.58)	0.042
Lymph node metastasis (−/+)	13/47	2.03 (1.01–4.09)	0.047	2.69 (1.51–4.81)	0.0008
Histological grade (0,1/2,3)	53/7	1.21 (0.48–3.07)	0.69		
Pre-treatment tumor volume (< 7000/≥ 7000) (mm^3^)	20/40	0.75 (0.42–1.35)	0.34		
Stromal ratio (High / Low)	32/28	1.83 (1.03–3.24)	0.038	1.89 (1.20–2.99)	0.0061
TNC (Low/High)	40/20	1.23 (0.66–2.31)	0.51		
POSTN (Low/High)	44/16	0.55 (0.28–1.08)	0.084	1.06 (0.67–1.68)	0.36
Ki-67 (Low/High)	24/36	1.21 (0.68–2.15)	0.51		

The left category is the reference for the hazard ratio

n indicates the number of participants, *HR* hazard ratio, *CI* confidence interval, *GnP* gemcitabine plus nab-paclitaxel, *GS* gemcitabine plus S-1, *PVR* portal vein resection

## Discussion

In this study, we focused on the biological modulation of the stroma by NAC and compared the effect of different NAC regimens on the stroma and its interaction with PDAC cells. Our findings demonstrated that patients with a low stromal ratio experienced rapid recurrence after curative resection, particularly liver metastasis. Furthermore, a low stromal ratio (i.e. the depletion of stroma more than cancer cells) by NAC, especially GnP, was positively correlated with high Ki-67, indicating that both the impairment of the stromal defense and hyperactivation of PDAC cells facilitate early liver metastasis in patients with BR PDAC.

PDAC is an extremely stroma-rich mass; almost 90% of the tumor is made of ECM consisting of a complex assembly of activated fibroblasts (myofibroblasts), pancreatic stellate cells, immune cells, and various matricellular proteins [21]. In addition to collagens, the ECM of PDAC is composed of TNC and POSTN, which we have previously identified as downstream targets of Prrx1, which fosters liver metastasis in PDAC [16]; therefore, they can be considered key molecules in tumor progression [22]. Here, we observed that NAC reduced the expressions of TNC and POSTN, accelerating liver metastasis; however, neither correlated with the stromal ratio, which was strongly correlated with liver metastasis. Understanding these paradoxical findings is necessary to elucidate the complex mechanisms of stromal biology. GnP is thought to reduce the tumor stroma, and the accompanying increase in vascularization caused by nab-paclitaxel facilitates the delivery of gemcitabine to PDACs [23]. Alvarez et al. showed that PDACs treated with GnP had a less abundant fibrillar collagen matrix [24]. We also observed that GnP decreased the stromal ratio and radiological tumor volume; however, the stromal ratio did not correlate with tumor shrinkage. These results suggest that the radiological response is not directly linked to the actual volume of cancer cells because PDACs are mostly composed of ECM.

To understand the effect of NAC on the balance of stroma and cancer cells, we examined the ratio of stroma to cancer cells in PDAC tissues. PDACs treated with GnP had a lower stromal ratio than those treated with GS. Together with the findings of previous reports [23, 24], these findings clearly indicate the chemotherapeutic effect of nab-paclitaxel in reducing both stromal volume and PDAC cells, whereas S-1 mainly has a cytotoxic effect against PDAC cells without modulating the stroma. It has been reported that patients with a high stromal ratio have a better prognosis than those with a low stromal ratio among patients with colon cancer, hepatocellular carcinoma, epithelial ovarian cancer, gallbladder cancer, and breast cancer [25–29]. Thus, to improve the outcome of PDAC patients, it would be needed not only to consider the radiological response for tumors but also to avoid decrease of the stromal ratio during NAC before surgery.

In this study, a low stromal ratio was associated with early liver metastasis in patients with BR PDAC treated with NAC. Animal model studies have suggested that stromal depletion approaches using genetic deletion of sonic hedgehog or a smoothened inhibitor can tip the balance toward tumor-promoting effects, resulting in highly proliferative and invasive PDACs [30, 31]. Similarly, Jiang et al. recently described that experimental stromal depletion with anti-lysyl oxidase like-2 antibody in an orthotopic PDAC mouse model led to lower tissue stiffness and accelerated tumor growth, resulting in diminished OS [32]. Indeed, several clinical trials have shown than depleting hyaluronan, a stromal component, failed to improve survival [13, 14]. In this study, a low stromal ratio was positively correlated with high Ki-67 in the NAC group but not in the UpS group, indicating increased aggressiveness of PDAC cells through the depletion of stroma modulated by NAC. A recent animal study demonstrated that deletion of type 1 collagen in myofibroblasts accelerates PDAC and diminishes OS [33]. Interestingly, lower type 1 collagen correlates with decreased T and B lymphocytes and increases immunosuppressive cells in mouse and human PDAC, indicating that a lower stromal ratio changes the TME of PDAC from tumor-promoting to tumor-inhibiting. Furthermore, Maurer et al. demonstrated two subtypes of tumor-associated stroma: the immune-rich subtype, which is related to immune-associated processes, and the ECM-rich subtype, which is related to ECM-associated pathways [34]. Survival analysis showed a trend toward a worse outcome in patients with the ECM-rich subtype. These experimental basic and clinical studies will help guide novel treatment strategies for PDAC to refine the optimal NAC regimen, including immunomodulation therapy.

There are inherent limitations in this study, such as information and referral biases due to the retrospective, single-center nature of the study. For example, the GS group had tended to have fewer pancreaticoduodenectomy and more cases of BR-A and arterial resection compared to the GnP group. This is because distal pancreatectomy with celiac axis resection has been often conducted from 2012 to 2016. Of note, there was no significant difference between the stromal ratio and the clinical factors that showed significant differences among the three treatment groups in Table 1. Furthermore, the number of participants was relatively small, and it may not be possible to note fine differences in the data. The alteration of cancer immunomodulation by NAC is needed to elucidate the mechanism of crosstalk between stroma and cancer cells in the TME of PDAC.

In conclusion, we demonstrated that the low stromal ratio induced by NAC increased early liver metastasis in patients with BR PDAC. Based on the results in this study, NAC-GnP and NAC-GS therapies would show chemotherapeutic effects against PDAC through the different mechanisms underlying the stromal remodeling. However, it is still difficult for individuals to decide the optimal NAC treatment for BR PDAC. A more in-depth understanding of the stromal and TME modifications by different NAC regimens will lead to better personalized preoperative treatment strategies in patients with BR PDAC.

## Supplementary Information

Below is the link to the electronic supplementary material.

Supplementary file1 (PDF 7062 KB)

## Data Availability

All the data generated in this study are included in this paper. The data presented in this paper will be available from the corresponding author upon reasonable request.

## Abbreviations

PDAC: Pancreatic ductal adenocarcinoma
BR: Borderline resectable
NAC: Neoadjuvant chemotherapy
TME: Tumor microenvironment
GnP: Gemcitabine plus nab-paclitaxel
GS: Gemcitabine plus S-1
RFS: Recurrence-free survival
CT: Computed tomography
BSA: Body surface area
IQR: Interquartile range
ROC: Receiver operating characteristic
TNC: Tenascin C
POSTN: Periostin
Gem: Gemcitabine
