# Supplementary material for: The effect of the low stromal ratio induced by neoadjuvant chemotherapy on recurrence patterns in borderline resectable pancreatic ductal adenocarcinoma
Source: Clin Exp Metastasis. 2022 Jan 9;39(2):311–22. doi: 10.1007/s10585-021-10142-7 (PMC8971157; doi:10.1007/s10585-021-10142-7)

Supplementary Table S1 Univariate and multivariate analysis of predictors of recurrence-free survival in the upfront surgery group

Clinico-pathological parameters	n	Univariate analysis		Multivariate analysis	
		HR (95% CI)	*p* value	HR (95% CI)	*p* value
Age (< 70 / ≥ 70) (years)	26 / 18	0.98 (0.51-1.88)	0.95		
Sex (M / F)	29 / 15	1.25 (0.64-2.42)	0.52		
PVR (− / +)	9 / 35	2.54 (0.99-6.53)	0.053	1.89 (1.16-3.06)	0.010
Arterial resection (− / +)	40 / 4	0.36 (0.09-1.49)	0.16		
Preoperative CA19-9 (< 70 / ≥ 70) (U/mL)	15 / 29	0.90 (0.47-1.75)	0.76		
Lymph node metastasis (− / +)	8 / 36	2.37 (0.92-6.12)	0.074	2.17 (1.24-3.80)	0.0068
Histological grade (0,1 / 2,3)	37 / 7	0.45 (0.16-1.27)	0.13		
Pre-treatment tumor volume (< 7000 / ≥ 7000) (mm^3)	24 / 20	1.29 (0.68-2.46)	0.43		
Stromal ratio (High / Low)	11 / 33	1.21 (0.57-2.58)	0.61		
TNC (Low / High)	18 / 26	1.60 (0.83-3.11)	0.16		
POSTN (Low / High)	24 / 20	1.20 (0.63-2.27)	0.58		
Ki-67 (Low / High)	27 / 17	1.25 (0.65-2.41)	0.50		

The left category is the reference for the hazard ratio.
n indicates the number of participants; HR, hazard ratio; CI, confidence interval; PVR, portal vein resection

Supplementary Figure legends

Title: The effect of the low stromal ratio induced by neoadjuvant chemotherapy on recurrence patterns in borderline resectable pancreatic ductal adenocarcinoma

Journal: Clinical & Experimental metastasis

Authors: Kenji, Kawahara MD; Shigetsugu, Takano MD, PhD*; Katsunori, Furukawa MD, PhD; Tsukasa, Takayashiki MD, PhD; Satoshi, Kuboki MD, PhD; Masayuki, Ohtsuka MD, PhD

Correspondence author:

Shigetsugu Takano MD, PhD

Department of General Surgery, Chiba University, Graduate School of Medicine

E-mail: stakano@faculty.chiba-u.jp

Supplementary Fig. S1. Kaplan-Meier curves according to preoperative chemotherapy and presence of liver metastases

(A) OS in the NAC and UpS groups. (B) OS according to liver metastasis in the NAC group. NAC, neoadjuvant chemotherapy; OS, overall survival; UpS, upfront surgery.

Supplementary Fig. S2. Images used to calculate the stromal ratio from Masson's trichrome-stained specimens

(A) Original image. (B) Image with the widest range of collagen extracted to light blue and

replaced with green. Images with the (C) second, (D) third, (E) fourth, (F) fifth most widely extracted collagens replaced with green. (G) Image of collagen with only the darkest blue staining extracted and replaced with green. (H) Image with red-stained cytoplasm extracted and replaced with green. The stromal ratio was equal to the sum of the green pixels in B, C, D, E, F, and G divided by the green pixels in H. (I) The optimal cut-off point for stromal ratio to predict survival by ROC curve analysis.

AUC, area under the curve; ROC, receiver operating characteristic.

Supplementary Fig. S3. Evaluation of tumor volume shrinkage and Evans grading

(A) The optimal cut-off point for tumor volume shrinkage to predict survival by ROC curve analysis. (B) Correlation between stromal ratio and Evans grade. (C) Correlation between treatment method and Evans grade.

AUC, area under the curve; ROC, receiver operating characteristic.

Supplementary Fig. S4. Evaluation of POSTN expression in BR PDAC specimens

(A) IHC analysis was performed using an anti-POSTN antibody, and patients were classified into two groups according to POSTN expression. Upper figure: High expression of POSTN. Lower figure: Low expression of POSTN. Scale bar: 200 μm. (B) RFS according to POSTN expression. (C) Correlation between POSTN expression and liver metastasis. (D) Correlation between treatment method and POSTN expression.

BR PDAC, borderline resectable pancreatic ductal adenocarcinoma; IHC,

immunohistochemistry; POSTN, periostin; RFS, recurrence-free survival.

Title: The effect of the low stromal ratio induced by neoadjuvant chemotherapy on recurrence patterns in borderline resectable pancreatic ductal adenocarcinoma

Journal: Clinical & Experimental metastasis

Authors: Kenji, Kawahara MD; Shigetsugu, Takano MD, PhD*; Katsunori, Furukawa MD, PhD; Tsukasa, Takayashiki MD, PhD; Satoshi, Kuboki MD, PhD; Masayuki, Ohtsuka MD, PhD

Correspondence author:
Shigetsugu Takano MD, PhD
Department of General Surgery, Chiba University, Graduate School of Medicine
E-mail: stakano@faculty.chiba-u.jp

Supplementary Fig. S1

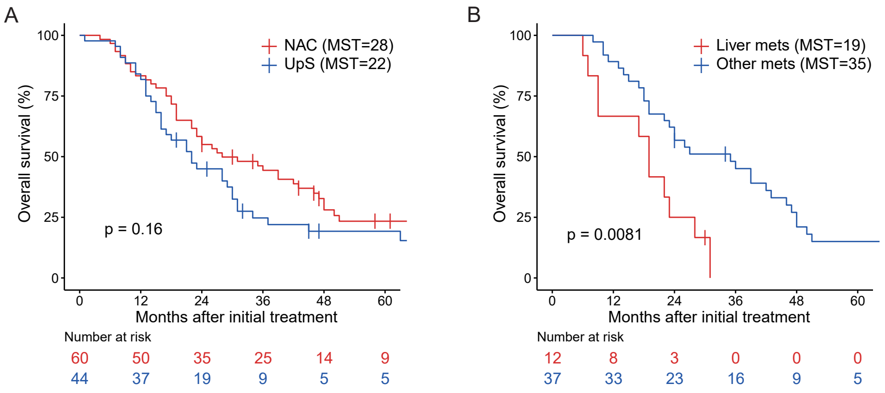

Title: The effect of the low stromal ratio induced by neoadjuvant chemotherapy on recurrence patterns in borderline resectable pancreatic ductal adenocarcinoma

Journal: Clinical & Experimental metastasis

Authors: Kenji, Kawahara MD; Shigetsugu, Takano MD, PhD*; Katsunori, Furukawa MD, PhD; Tsukasa, Takayashiki MD, PhD; Satoshi, Kuboki MD, PhD; Masayuki, Ohtsuka MD, PhD

Correspondence author:
Shigetsugu Takano MD, PhD
Department of General Surgery, Chiba University, Graduate School of Medicine
E-mail: stakano@faculty.chiba-u.jp

Supplementary Fig. S2

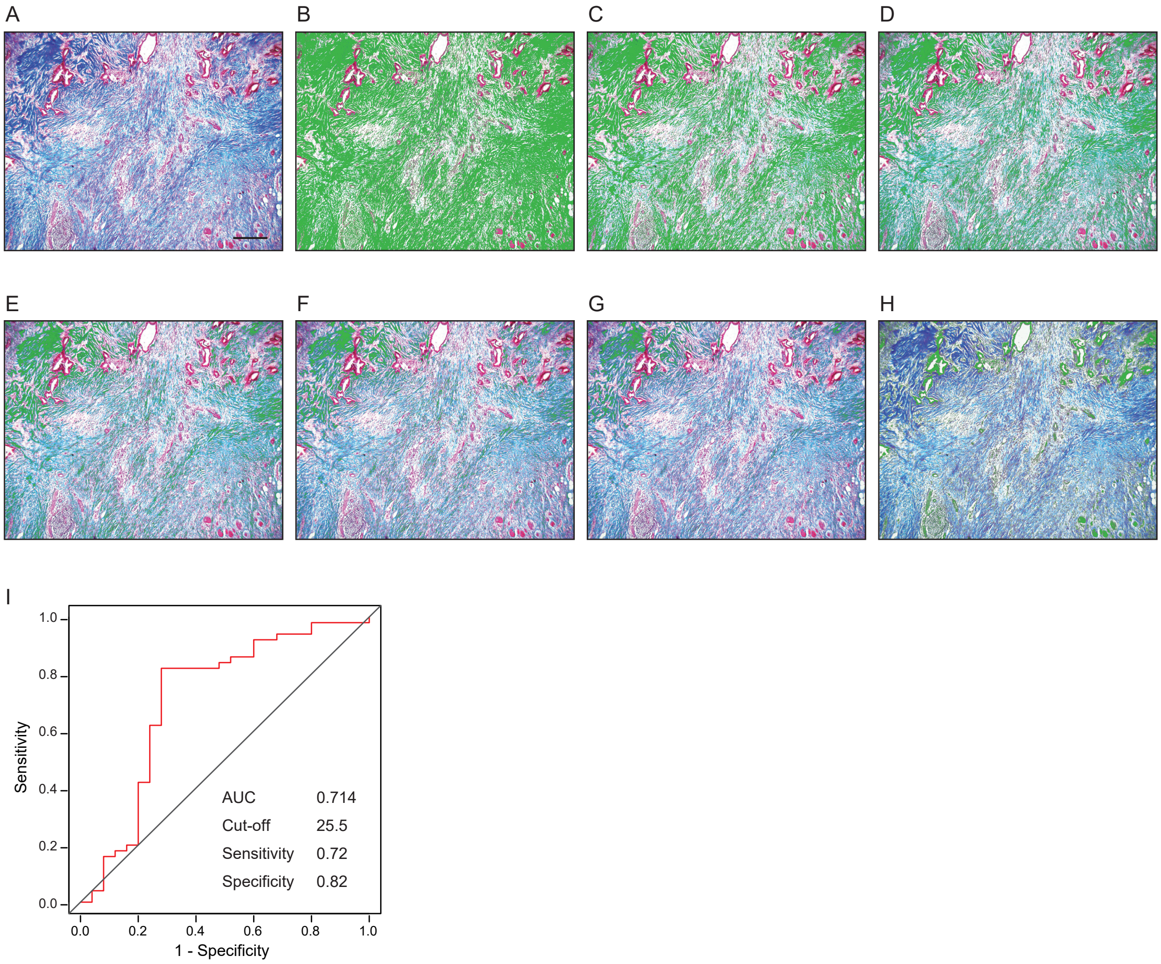

Title: The effect of the low stromal ratio induced by neoadjuvant chemotherapy on recurrence patterns in borderline resectable pancreatic ductal adenocarcinoma

Journal: Clinical & Experimental metastasis

Authors: Kenji, Kawahara MD; Shigetsugu, Takano MD, PhD*; Katsunori, Furukawa MD, PhD; Tsukasa, Takayashiki MD, PhD; Satoshi, Kuboki MD, PhD; Masayuki, Ohtsuka MD, PhD

Correspondence author:
Shigetsugu Takano MD, PhD
Department of General Surgery, Chiba University, Graduate School of Medicine
E-mail: stakano@faculty.chiba-u.jp

Supplementary Fig. S3

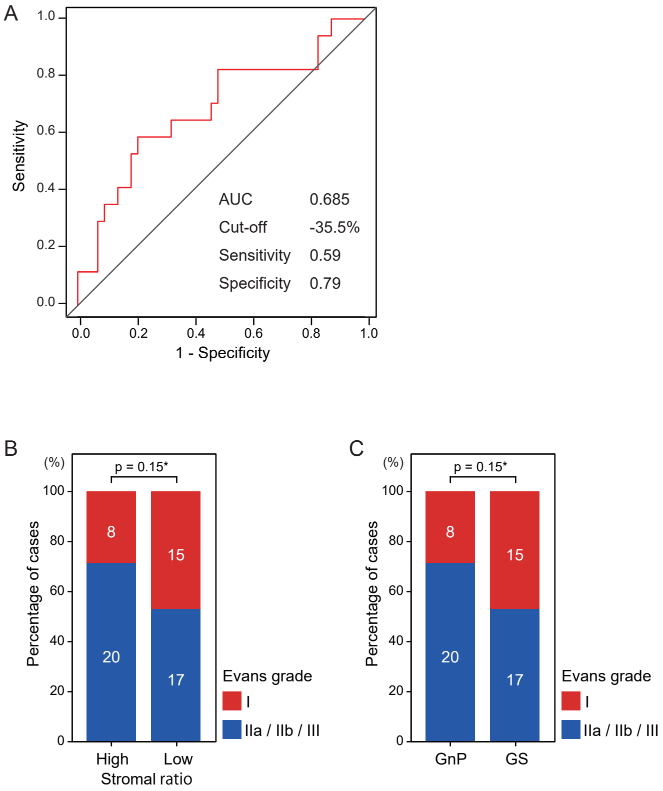

Title: The effect of the low stromal ratio induced by neoadjuvant chemotherapy on recurrence patterns in borderline resectable pancreatic ductal adenocarcinoma

Journal: Clinical & Experimental metastasis

Authors: Kenji, Kawahara MD; Shigetsugu, Takano MD, PhD*; Katsunori, Furukawa MD, PhD; Tsukasa, Takayashiki MD, PhD; Satoshi, Kuboki MD, PhD; Masayuki, Ohtsuka MD, PhD

Correspondence author:
Shigetsugu Takano MD, PhD
Department of General Surgery, Chiba University, Graduate School of Medicine
E-mail: stakano@faculty.chiba-u.jp

Supplementary Fig. S4

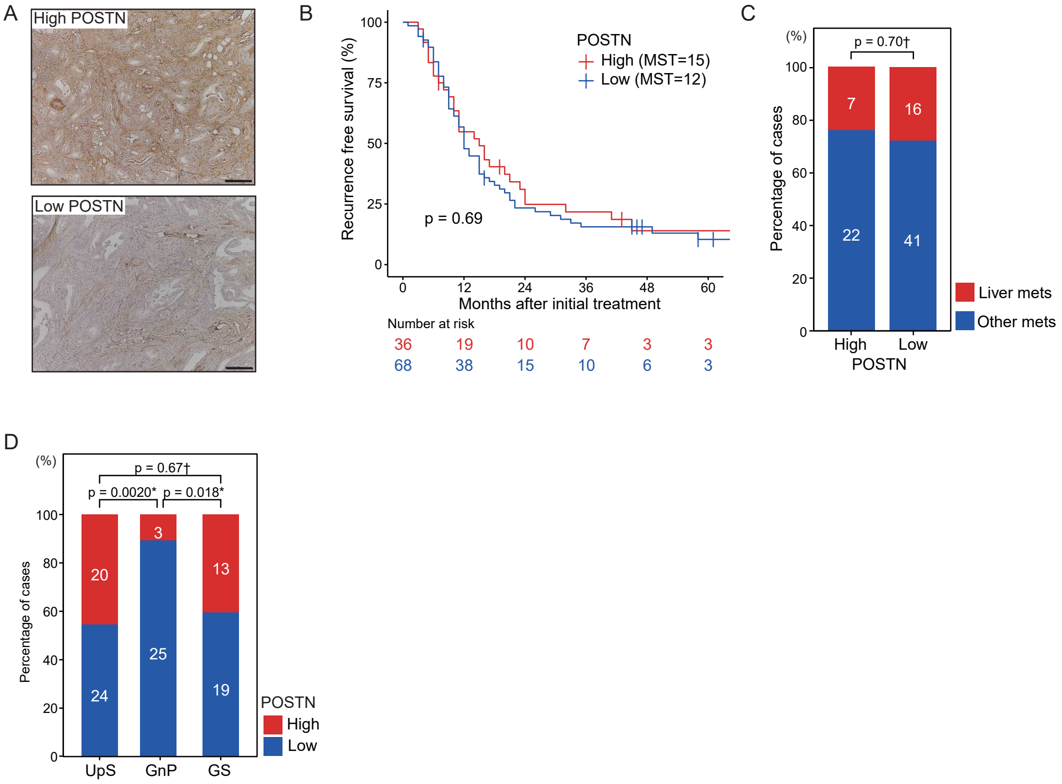

Supplement: Supplementary file 1 — Supplementary file1 (PDF 7062 KB) [file 10585_2021_10142_MOESM1_ESM.pdf]
